# A near-synoptic survey of ocean microplastic concentration along an around-the-world sailing race

**DOI:** 10.1371/journal.pone.0243203

**Published:** 2020-12-08

**Authors:** Toste Tanhua, Sören B. Gutekunst, Arne Biastoch

**Affiliations:** 1 GEOMAR Helmholtz Centre for Ocean Research Kiel, Kiel, Germany; 2 The Ocean Race, Alicante, Spain; 3 SCG Science Consulting–Dr.-Ing, Gutekunst, Koblenz, Germany; 4 Christian-Albrechts-University Kiel, Kiel, Germany; Evergreen State College, UNITED STATES

## Abstract

Litter and plastic pollution in the marine environment is of major concern when considering the health of ocean ecosystems, and have become an important focus of ocean research during recent years. There is still significant uncertainty surrounding the distribution and impact of marine plastic litter on ocean ecosystems, and in particular on the nano- and microplastic fractions that are difficult to observe and may be harmful to marine organisms. Current estimates of ocean plastic concentrations only account for a small fraction of the approximated 8 million tons of plastic litter entering the oceans on an annual basis. Here, we present the distribution of 100–500 μm microplastic particles within the ocean mixed layer, covering a significant fraction of the ocean, in a near-synoptic survey. During *The Ocean Race* 2017/2018 edition (formerly known as *Volvo Ocean Race*), two yachts served as ships of opportunity that regularly took samples of microplastics on a regular schedule during their circumnavigation. This effort resulted in information on microplastic distribution along the race track in the ocean’s upper, well-mixed, layer. We found concentrations ranging from 0–349 particles per cubic meter, but with large spatial variability. There was a tendency toward higher concentrations off south-western Europe and in the southwest Pacific, and indications of long-range transport of microplastic with major ocean currents.

## Introduction

The ocean is on the receiving end of plastic litter and pollution from human populations. Although the majority of litter is disposed of on land, streams and rivers still carry plastic waste into the ocean. It is estimated that roughly 8 million tons of plastic litter enters the ocean on an annual basis [1]. Currently, the fate of the majority of this plastic is unknown. Only a small fraction of the plastic input can be accounted for [2] by scaling up observational estimates to a global scale. Although it is generally assumed that a large fraction of the plastic waste ends up sinking to the bottom of the ocean, there are few estimates of the scale of this pathway and no inventory of plastic in or upon the sediment. However, it is clear that various processes tend to break up large plastic litter to smaller particles that can stay suspended in the water column for a long time. The effect of plastic litter on marine life and ecosystems is a topic of intense research; it is clear that plastics can accumulate in the ingestion systems of larger animals [e.g. 3,4], leading to malnutrition or death [5]. In addition, studies have shown that ghost nets can trap animals [6,7]. As the plastic particles get smaller, the potential for uptake by smaller organisms such as zooplankton and phytoplankton is obvious [8–11]. The long residence time of plastic in the environment also leads to accumulation of plastic particles in the food-chain, with an impact on humans through consumption of seafood [12,13].

Plastics constitute a large range of materials composed of a variety of polymer types, with microplastics typically defined as particles between 1 μm and 5 mm in size [14,15]. Furthermore, microplastic comes in many different forms, such as textile fibres made of nylon or particles e.g. used in cosmetics or for plastic article production. There are two different, principal sources of microplastics in the marine environment: primary and secondary sources. Primary sources are defined as small pellets the size of millimetres to centimetres, commonly used, for instance, in extrusion or moulding of thermoplastic polymers. Secondary microplastics are defined as particles or fibres formed during the decomposition of polymers from, e.g. fishing gear or unrecycled polymers. Decomposition is mediated through UV-irradiation, which makes most polymers brittle, as well as wear and friction that creates smaller and smaller pieces, which finally fall into the microplastics category. Most particles eventually decompose into even smaller particles called nanoplastic particles. Nonetheless, polymers generally withstand eroding or naturally decomposing mechanisms well, contributing to the accumulation of plastic litter in nature [16], although the decomposing rates in the marine environment is often poorly constrained [17].

The distribution of marine plastics in the water column, or at the sea surface, is partially known from a number of studies and reviews [2], although different sampling and measurement techniques make direct comparisons difficult [18]. A study of Cózar, et al. [19], for instance, combines data from a circumnavigation and modelled distribution of microplastics, finding enhanced concentrations in the subtropical gyres, in general agreeing with model predictions. The purpose of the present study is to provide a snapshot of microplastic distribution in the global ocean mixed layer, sampled and measured in a coherent and near-synoptic manner. Here we present observations from two race yachts participating in a race around the world, the 2017/18 edition of *The Ocean Race* (formerly known as the *Volvo Ocean Race*). Although this study does not cover all ocean sub-basins, we provide a near-global perspective on pelagic microplastic distribution, utilizing a novel sampling platform and a fast throughput measurement system.

## Materials and methods

For this survey, we utilized two ocean-going race yachts competing in the 2017/2018 edition of *The Ocean Race* as observation platforms. We installed sampling equipment on two of the 65’ one-design yachts known as Volvo Ocean 65s, namely *Turn the Tide on Plastic* and *AkzoNobel*. The race started in Alicante, Spain and ended in den Hauge, Netherlands after racing around the world. In addition, we have included data from the pre-race and post-race legs to and from Lisbon, Portugal. The race had port-stops in Lisbon (Portugal), Cape Town (South Africa), Melbourne (Australia), Hong Kong, Auckland (New Zealand), Itají (Brazil), Newport (Main, USA), Cardiff (UK), Gothenborg (Sweden), and The Hauge (Netherlands). No permission for marine scientific research is required for areas beyond national jurisdiction (i.e. the high seas). The study did not include any endangered species. The boat captains, responsible for technical equipment on the yachts, were adequately trained to minimize environmental impacts caused by themselves to their surroundings and were educated on the omnipresence of microplastic particles and the risks of contamination. New filters were positioned next to the sample holder before opening the latch to reduce air exposure. The captains immediately stored the previous samples in aluminium bags, simultaneously installing the new filters in order to minimize exposure time to ambient air. No plastic gloves were used during the process. The time needed to change the filters of one sample in this procedure took less than one minute, on average, and never exceed three minutes. Although the *Turn the Tide on Plastic* took samples throughout the whole race, *AkzoNobel* only collected samples for half of the race, after the port-stop in Auckland. See supplementary material for a detailed list of sampling positions.

During the race, a total of 96 microplastic samples were taken by filtering seawater through up to three stainless steel filters, although only those within the 100–500 μm size fraction were analysed (S1 Table in S1 File and below). These filters were securely packed and transported to the lab in Kiel to be measured for their microplastic concentration after sampling. The water supply was from one of two inlets located at each side of the yachts bottom; the leeward intake was always used to avoid air contamination in the system. Some water for the yacht’s water maker supply was diverted to the filtration system and an analytical system that measured salinity, temperature, chlorophyll a and partial pressure of carbon dioxide (pCO_2_). We will not discuss the pCO_2_ observations in this paper, but we note that the pCO_2_ instrument (OceanPack™Race by SubCtech GmbH) did continuously log the position during sampling, as well as the volume of sampled water from a flowmeter located at the filtration unit. The filters were exchanged every two days on long ocean legs, and every day on shorter legs–a practice that reduced the workload for the crew and limited the extra weight of filters the yachts had to carry to remain competitive in the race. The sampling interval typically lasted slightly less than one hour every 20 hours (the timing decided by the need for fresh water for the yachts), and most filters were sampled during one or more of such charging cycles. This resulted in a wide range of sampling-times and volumes (see S1 Table in S1 File for details). The samples were typically taken 0.5–1.5 meter below the sea surface, although this is dependent on sea-state and heel, being representative of the mixed-layer microplastic concentration, rather than the microplastics floating on the surface.

### Sampling and sample pre-treatment

All glassware was cleaned prior to its use with HPLC grade water. Precautions were taken by wearing 100% non-synthetic clothes, use of freshly washed hands without rubber gloves, lab surfaces were cleaned with HPLC grade water and nonabrasive wipes (Kimtech Science™ Kimwipes™ Delicate Task Wipes). The sampling equipment on-board the yachts consisted of sets of three custom-built stainless-steel filters with an opening diameter of 44 mm and mesh-size of 30, 100 and 500 μm. Since we found few particles in the large (500 μm) filters due to insufficient sampling volume, we will not consider these particles here. Similarly, we were not able to quantify the amount of microplastic particles on the smallest filters (<100 μm) reliably, so we will not consider those particles in this study. The extraction and measurement of the filter cake was conducted according to [20] but with the following changes: The filter was put into a glass beaker with SDS-solution (1%, Sigma-Aldrich solid) and double distilled water (Servoprax Aqua bidest <0.5 μS/cm); The sample was extracted from the filter with ultra-sonication at 30°C for 15 min and another 5 min with the filters turned upside-down; The sample was concentrated by low vacuum filtration onto a wet cleaned nylon filter (Millipore, 10 μm) and vigorously washed (three times each with 10 mL CarlRoth Rotisolv HPLC grade); The filter cake was washed from the filter into a glass-beaker with 25 mL HPLC grade water and washed with an additional 5 mL until complete dissolution of the filter cake into the sampling glass was achieved.

### Microplastic raman measurements

For the analysis and detection of microplastic particles, we used a flow-through system connected to a Raman laser (CivilLaser DPSS Laser, 532 nm, 1000mW) and detection on a Hamamatsu MiniSpectrometer (C13555MA). Prior to analysis of the samples, the instrumentation was cleaned with distilled water at a flow of 48 to 90 mL/h for 15–30 minutes. The Raman laser was switched on at least 3 min before measurement and analysis could commence after equilibration to the reference spectra of pure water after ~6 minutes. The sample in the glass-beaker was transferred into a capillary by a peristaltic pump at a flow of at least 40 mL h^-1^. A magnetic stirrer (Roth M3, speed setting 2.5, rod shaped stirrer) was used for homogenization of the samples with the Teflon inlet mounted against the stirring direction. Before the end of the sample volume was reached, an additional 5 mL of HPLC grade double distilled water was added and repeated when necessary, to secure the complete transfer of the sample into the flow-through Raman system. With the given flow rate and capillary dimensions, one particle passing the laser focal point provided one to three spectra. After each sample, the instrumentation was cleaned with additional HPLC grade water to assure that no microplastic particles remained in the tubing.

The spectra from the analytical procedure were pre-analyzed by a Gaussian algorithm for the counting of events (i.e. the passing of a particle in the cell) compared to the spectra with no particles (i.e. baseline) with the peak indicating the presence of microplastics. Presence of microplastic was indicated by a peak in the scattered wavelengths between 627–632 nm when the detected intensity was more than 300% higher than its baseline. These events were compared with reference spectra from measured polymer particles and considered as microplastic particles analysed he same way. We estimate one microplastic particle for 500–5000 other particles (biological or mineral). Due to the specialized Raman design and laser interaction times, our measurements had sufficient signal to noise ratio for particle detection. Fibres were not detected as their measured intensity was below the 300% signal intensity compared to the baseline due to their shape and interaction times. In order to estimate the possible number of false positives, we analyzed blanks from the lab environment. As described by Lenz, et al. [20], petri-dish samples (n = 6) filled with bi-distilled water exposed to the laboratory's environment were used to sample for airborne fragments during sample preparation of sea water samples. These samples usually contained less than five fibres and less than one particle each, which is below the instrument setup resolution limit due to their microns-wide diameter which lead us to conclude to less than one false positive per sample. Therefore, no further uncertainty due to the laboratory environment was included.

### Statistical analysis

The recovery rate and precision of the instrument setup for microplastic analysis was evaluated through repeated measurement of known quantities of microplastic particles, as well as repeated sample measurements. From repeat measurements (N = 6) of 25 manually counted particles with a size of >100 μm within a microplastic particle solution made from PA, we found an averaged recovery rate of >92% (α = 0.08). Similarly, repeated measurements (N = 6) of samples indicated an uncertainty in particle counts of less than one particle count, so that no adjustment of the particle count error was necessary. The final total counts of measured microplastic particles were corrected by 8% to account for the less than 100% recovery rate. The largest source of uncertainty in the observations is related to counting statistics, since the sample volume was in general in the order of less than ¼ m^3^ (see S1 Table in S1 File). Depending on the combination of sampling volume and particle density, the uncertainty of the observations is approximately 20% (S2 Fig in S1 File).

## Results and discussion

### Results

**Fig 1:**
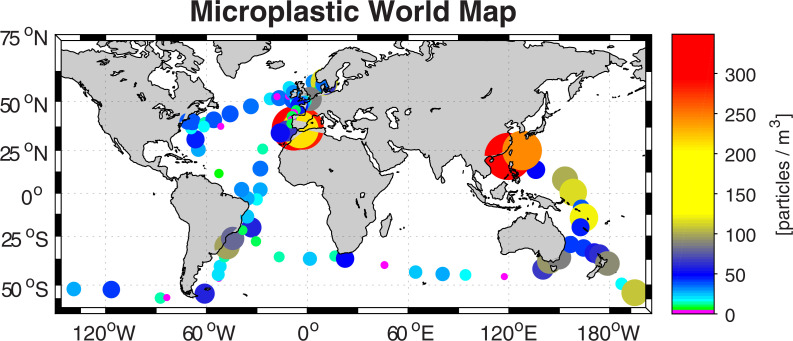
The spatial distribution of the measured microplastic concentration in units of particles per cubic metre, as indicated by the colour and size of the dots. Note that each observation represents an average over potentially several sampling positions of the same filter, see S1 Table and S2 Fig in S1 File.

We obtained data from 96 filters (see S1 Table in S1 File) over the entire race track, which allowed us to draw a global map of microplastic concentrations in the mixed layer (Fig 1). An alternative view of the results is presented in S2 Fig in S1 File, where all water sampling locations are indicated. The particles measured in this study are likely close to neutrally buoyant since we sample below the surface, in the upper part of the water column. However, during rough seas floating particles are likely to be pushed down below the surface by wave action, and the yachts are “bouncing” through the waves in such a way that floating particles could end up in our sampling system.

We found microplastic particles in almost all samples (93 out of 96 samples), independent of how remote the sampling location was from land. The average abundance of microplastic particles in our survey is 50 particles m^-3^, with the microplastic concentration ranging from 0 to ~350 particles m^-3^. The highest concentrations were observed in the West Tropical North Pacific Ocean (the South China Sea/ West Philippine Sea, 243–349 particles m^-3^ in two samples, S1 Table in S1 File). High concentrations were found as well in the Western Mediterranean and the Gulf of Cadiz, where concentrations consistently ranged from 180 to 307 particles/m^3^ (S1 Table in S1 File) in four samples. The western tropical Pacific had generally high concentrations with an average of 86 particles m^-3^ for legs 4 and 6 (Melbourne–Hong Kong–Auckland), although higher concentrations were found on the southbound leg (see discussion below).

The North Atlantic Ocean (samples north of the equator), on the other hand, had a relatively modest abundance of microplastics with an average of 25 particles m^-3^ (S1 Table in S1 File). This is similar to the South Atlantic Ocean’s average of 29 particles m^-3^, which was mainly due to somewhat higher concentrations along the coast of South America. Low concentrations were found in the Southern Ocean samples (south of 35°S) where the average abundance was 18 particles m^-3^ for open ocean samples. The average Southern Ocean concentration when including samples close to South America, Australia, New Zealand and Africa increases to 34 particles m^-3^, as a consequence of higher concentration of particles close to continents and in major ocean current systems.

The results, with concentrations in the range of a few tens of particles m^-3^ in offshore waters versus more than one hundred particles m^-3^ in areas close to the continents, reveal that there is a general tendency for the microplastic concentration to be higher close to the continents. Similarly, there is a tendency to find higher concentrations of microplastic in locations associated with major ocean currents, such as in the Agulhas Current regime off South Africa or in the Antarctic Circumpolar Current/Flinders Current south of Australia. We have two samples in the vicinity of the Cape of Good Hope, one with 12 ± 13 and the other with 46 ± 12 particles m^-3^, both at a similar distance from the continent. Lagrangian experiments (see supplementary material for more information and animations) exploring their potential origin by simulating the backward drift of virtual plastic particles within an operational ocean model demonstrate the influence of the Agulhas Current system on these two sample locations. The Agulhas Current flows southward along the African continent in the Indian Ocean [21]. South of Africa, it abruptly turns back into the Indian Ocean and releases part of its warm and saline water of tropical origin into the South Atlantic. This ‘Agulhas leakage’ is important for global climate and leaves the waters southwest off the Cape of Good Hope warmer than the surrounding Atlantic [22]. The simulations show that virtual plastic particles arriving through the Agulhas Current are more highly concentrated in the fan of Agulhas leakage. In contrast, the other sampling location further west is outside this direct influence, hence contains less microplastic particles.

We do not expect that 96 samples from around the world can be more than a snapshot of the global microplastic distribution, especially since the microplastic concentrations tend to exhibit high spatial and temporal variability. To illustrate this, we utilize the fact that one area was sampled twice within one month: the leg from Melbourne to Hong Kong and then back to Auckland, representing a quasi-repeat observation of the same general ocean area. Note that we do not have continuous sampling, and each sample is an integrated sample over several hours of sampling (S2 Fig in S1 File). These measurements in the western tropical Pacific Ocean reveal patchy and variable distribution patterns, with sampling points close to each other showing significantly different concentrations. For instance, observations during legs 4 and 6 in the area between 4° and 14° S varies between 20 and 122 particles m^-3^ (Fig 1). The difference in concentration between the two repeats of a similar track is useful for understanding the variability and the potential bias of the limited sampling. The differences are particularly large in the western part of the sections close to the Asian continent, and is likely related to particles being preferentially transported with the major current systems, such as the Kuroshio Current and the North Equatorial Current. Whether or not the samples were taken in the current is likely to produce significant differences in concentration. This is to say that any observation of microplastic in the upper ocean will be a snapshot of the concentration at one time at one occasion only and might, or might not, be representative for that particular area. The issue can, obviously, in general terms be solved by a more frequent (in space and time) observing scheme. This does not undermine the results in this study, but caution should be taken on putting too much emphasis on a single observation. The large, and hard-to-quantify, variability in microplastic distribution is a by far the largest reason for uncertainty compared to the well-quantified uncertainty of the measurements themselves.

### Difference in sampling

In order to explore how our results compare to previous studies, we must examine how differences in sampling techniques and schemes complicate the comparison. Firstly, we note that differences in sampling techniques and schemes complicate a direct comparison in many cases. For instance, the subsurface sampling used in this study is different from most surveys of surface microplastic distribution that typically use trawls (e.g. manta trawls) to collect samples at the sea-surface and the top few decimetres of the mixed layer. As demonstrated by [23] and modelled by [24], it can be expected that the subsurface microplastic concentration is lower by at least a factor of two compared to surface samples, though still highly dependent on a number of factors such as wave action, wind speed, and density and size of the microplastic particles. It should also be noted that the exact sampling positions were dictated by the sailor’s urge to find favourable wind and current conditions, not by potential hot-spots for microplastic in the ocean. Similarly, the sampling intervals were solely driven by the yacht’s need to run the desalination unit. For instance, while the high concentration of plastic in the subtropical gyres is well known [e.g. 25], they are areas of typically low winds and are generally avoided by the yachts. It is thus possible that our results are biased towards zones outside of the aggregation areas in the gyres.

Our sampling system was similar to that of Desforges, et al. [26], although our sampling was shallower (1–2 meters) than theirs (4 meters depth). The sampling method from Lusher, et al. [27] is also similar to ours, with the main difference that their filters were treated on-board, the sample was collected in a glass beaker and stored in glass microfiber paper for subsequent analysis in the lab. The study by Cózar, et al. [19] uses data from a circumnavigation during the Malaspina expedition, combined with other data. The data was collected from surface net trawls, the particles were larger than this study, and the data is presented in terms of mass km^-2^, making the results difficult to directly compare to ours. Kanhai, et al. [28] used a sampling method similar to ours, although the analytics in the lab differ. An important difference is the size of particles considered, and the fact that they did include fibers in the count (which our method did not).

Most studies report on surface concentrations as particles km^-2^, which is different from the volume-based concentrations of the mixed layer abundance. For the purposes of a first order estimate, we assume that the ocean’s mixed layer is 10 meters thick, which is a reasonable number for summer months [e.g. 29], although ocean mixed layer depth is highly variable in time and space. Our average of 50 particles m^-3^ (~5x10^^8^ particles km^-2^) is relatively high compared to, to the compilation by van Sebille, et al. [30], where a large number of surface trawl studies were reviewed. The logarithmic scale of their overview map spans from 10 to 10^7^, indicating that the number of particles found in this study is on the high range of previously reported microplastic abundances. One explanation for this difference is that this study reports on the 0.1–0.5 mm size fraction of plastic particles, which is in the lower range of the mesh size for surface trawl efforts. Thus there is likely a systematic bias in the particle concentration observed for the two different sampling methods. There is also evidence for temporally increasing concentrations of plastic in the ocean: Ostle, et al. [31] found a significant increase in the plastic entanglement of continuous plankton recorder (CPR) tows from 1937 to 2016. Therefore, it is possible that our field work during 2017/18 is likely to encounter a larger abundance of plastic.

### Comparison to previous published microplastic distributions

It is interesting to compare the data in this study to the compilation of existing data, and interpolated maps guided by models [e.g. 30]. In a compilation of data from almost 12,000 manta trawls [2,30], it is obvious that large parts of the ocean are under-sampled, or not sampled at all, for microplastics. This study adds data to some of the poorly sampled areas, in particular the Southern Ocean. Direct comparison to observations is possible in the Atlantic Ocean where our observations are roughly consistent with the generally higher concentrations in the South Atlantic (noting the difference in magnitude of particle counts as mentioned above), and in the vicinity of the North Atlantic subtropical gyre (noting that our observations cover that area poorly). Similarly, the study of Cózar, et al. [19] collected marine debris from one expedition around the world (the Malaspina expedition), and is in that sense comparable to our results, though most parts of the routes did not overlap. Whereas there is some consistency in the Atlantic between the surveys (taken 7 years apart), there are differences in the area south of Australia where we have high concentrations in comparison to the Malaspina data. A similar picture emerges in the tropical Pacific, although our data are located further west. Kanhai, et al. [28] reports on microplastic concentrations of 0–8 particles m^-3^ in a north-south section of the Atlantic Ocean, which is at the lower end of our measurements. This can potentially be explained by the larger size fragments considered in their study and the fact that it was carried out 2 years earlier than ours. Desforges, et al. [26] on the other hand found significantly higher concentrations of microplastics than in both this study and most other studies. This could possibly be due to different methods used to analyze the samples in the lab, the sample depth, or it could be related to high concentrations in the Northwest Pacific Ocean. The experiment by Lusher, et al. [27] found fewer particles in the Northeast Atlantic Ocean than in our study (0–22.5 particles m^-3^), which can be explained by the difference in analyzed size fraction: they analyzed the 250–1000 μm fraction, which is larger than in our study and therefore may have fewer particles.

These patterns are also observed by the models in van Sebille, et al. [30]. The models tend to reproduce the observations of elevated microplastic concentrations in the Mediterranean Sea and the low concentrations south of roughly 40–50°S (except for a few regions, e.g. SE of New Zealand). Note again that our data are from the area south of the sub-polar gyres, i.e. outside of the major accumulation areas.

The models are also consistent with the high concentrations we find off Asia, but do not quite represent the high concentrations we find in the tropical West Pacific Ocean. The models predict high concentrations in the Tasman Sea, while our data shows variable concentrations in this region.

## Conclusions

In this study we present a near-synoptic (within 9 months) study with near-global coverage of microplastic abundance, sampled and measured in a consistent manner. The survey confirms that microplastics are ubiquitous in the ocean, even at locations far from land. The data set also shows large temporal and spatial variability in the areas sampled for this study, with higher abundances in the vicinity of continents, and particularly high abundances in the Mediterranean Sea and South China Sea. It also shows particularly high abundances in the western tropical Pacific Ocean. Importantly, this data set indicates high tempo-spatial variability, calling for intensified sampling efforts using community agreed upon methods to facilitate a global compilation.

The fact that the samples size fraction and analytical methods for measurements are different for most studies that examine the subsurface concentration of microplastic makes it difficult to directly compare results. It also seems likely that the temporal and spatial variability of microplastics is high so that direct comparisons always will be challenging. The data reported here, and the difficulty in comparing to previous studies, point to the challenge in the emerging field of microplastic research. It is important for the scientific community to agree on common protocols for sampling, measurements, reporting etc. to facilitate an increased understanding of global microplastic distribution [15,18,32].

We show that using unconventional observing platforms (in this case ocean going race yachts) to take microplastic samples is feasible, and offers the potential to greatly increase the sampling and observing effort needed to adequately map ocean microplastic distribution. Thanks to their unique routes, we demonstrate that race yachts used as sampling platforms show good potential for providing observations useful to assess the distribution of microplastic.

## Supporting information

S1 File (DOCX)
