# Supplementary material for: A near-synoptic survey of ocean microplastic concentration along an around-the-world sailing race
Source: PLoS One. 2020 Dec 8;15(12):e0243203. doi: 10.1371/journal.pone.0243203 (PMC7723278; doi:10.1371/journal.pone.0243203)
Supplement: S1 File — (DOCX) [file pone.0243203.s001.docx]

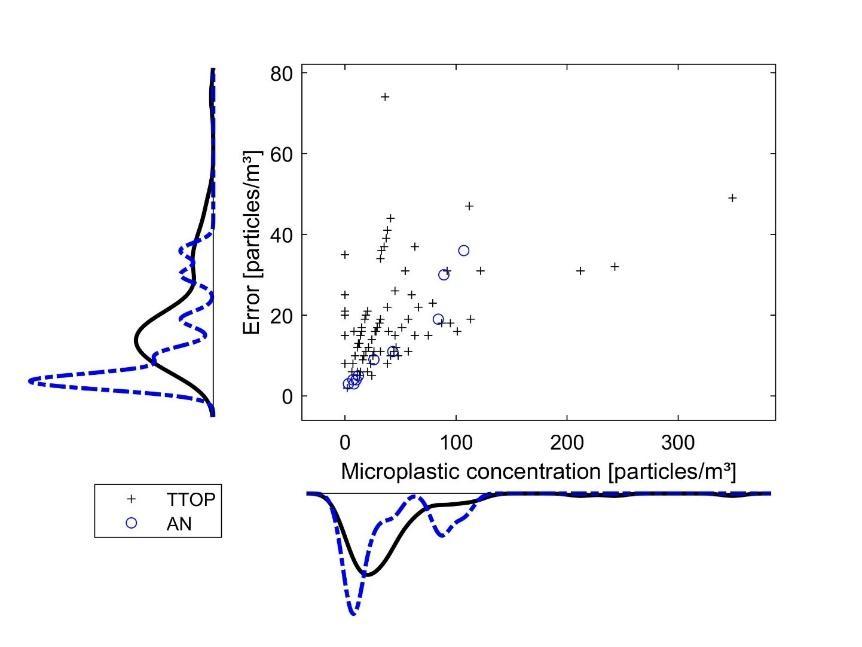


**Figure S1.** Error histogram of the microplastic samples; the x-axis refers to the measured content of microplastic for each sample, and the y-axis shows its uncertainty calculated from the statistical error analysis. The lines outside of the plot is the density function of the data: Turn the Tide on Plastic (TTOP, black cross, solid line); AkzoNobel (AN, blue circle, dashed line).

**Data and materials availability:**

NOAA NCEI Accession 0170967 including further data as pCO_2,_ sea surface temperature, salinity and meteorological data of all competing race yachts:

<https://data.nodc.noaa.gov/cgi-bin/iso?id=gov.noaa.nodc:0170967>

EMODnet:

<https://www.emodnet-ingestion.eu/submissions/submissions_details.php?menu=39&tpd=232&step=0103_001volvo%20ocean%20race>

File download microplastic data:

<https://cloud.emodnet-ingestion.eu/index.php/s/e16i2oD4jKGvPxg>

| **Table S1.** **Microplastic** **measurements and their averaged locations.** | | | | | | | |
| --- | --- | --- | --- | --- | --- | --- | --- |
| **Avg. Lat** | **Avg. Lon** | **Year / Month** | **Yacht / LEG** | **Filter volume [L]** | **Microplastic [particles/m³]** | **Distance [km] / Time [min]** | **Region** |
| 37,32 | -7,18 | 2017 / 10 | TTOP  0 | 1364 | 180 ± 16 | 496 / 2878 | Mediterranean  Atlantic Ocean |
| 36,88 | -3,27 | 2017 / 10 |  | 1031 | 307 ± 27 | 444 / 1647 |  |
| 37,84 | -0,83 | 2017 / 10 |  | 436 | 280 ± 24 | 139 / 641 |  |
| 36,12 | -4,25 | 2017 / 10 | TTOP  1 | 191 | 212 ± 31 | 56 / 113 |  |
| 35,39 | -12,18 | 2017 / 10 |  | 110 | 63 ± 15 | 56 / 156 |  |
| 34,39 | -16,06 | 2017 / 11 | TTOP  2 | 78 | 51 ± 17 | 119 / 178 | Atlantic Ocean |
| 25,68 | -26,53 | 2017 / 11 |  | 189 | 11 ± 06 | 166 / 351 |  |
| 14,51 | -27,90 | 2017 / 11 |  | 126 | 32 ± 11 | 103 / 194 |  |
| 2,02 | -27,97 | 2017 / 11 |  | 73 | 27 ± 16 | 85 / 102 |  |
| -3,70 | -30,07 | 2017 / 11 |  | 128 | 16 ± 09 | 162 / 307 |  |
| -20,13 | -33,22 | 2017 / 11 |  | 158 | 57 ± 11 | 110 / 220 |  |
| -27,92 | -30,52 | 2017 / 11 |  | 108 | 9 ± 10 | 71 / 174 |  |
| -36,25 | -16,72 | 2017 / 11 |  | 89 | 11 ± 12 | 85 / 127 |  |
| -37,23 | 1,58 | 2017 / 11 |  | 411 | 24 ± 05 | 145 / 703 |  |
| -35,81 | 16,43 | 2017 / 11 |  | 80 | 12 ± 13 | 59 / 120 |  |
| -37,15 | 22,51 | 2017 / 12 | TTOP  3 | 132 | 46 ± 12 | 91 / 187 | Indian Ocean  Southern Ocean |
| -40,42 | 45,95 | 2017 / 12 |  | 483 | 2 ± 02 | 155 / 830 |  |
| -43,90 | 64,49 | 2017 / 12 |  | 95 | 21 ± 12 | 160 / 234 |  |
| -44,82 | 80,57 | 2017 / 12 |  | 122 | 25 ± 10 | 213 / 336 |  |
| -45,29 | 94,16 | 2017 / 12 |  | 209 | 14 ± 06 | 195 / 334 |  |
| -46,07 | 117,29 | 2017 / 12 |  | 126 | 0 ± 08 | 270 / 432 |  |
| -42,35 | 140,33 | 2017 / 12 |  | 61 | 66 ± 22 | 63 / 122 |  |
| -38,51 | 144,16 | 2017 / 12 |  | 43 | 92 ± 31 | 23 / 68 |  |
| -36,47 | 149,86 | 2018 / 1 | TTOP  4 | 92 | 87 ± 18 | 93 / 181 | West Pacific Ocean |
| -29,63 | 156,70 | 2018 / 1 |  | 77 | 39 ± 16 | 58 / 107 |  |
| -12,42 | 161,98 | 2018 / 1 |  | 246 | 20 ± 06 | 157 / 564 |  |
| -4,57 | 161,41 | 2018 / 1 |  | 172 | 23 ± 08 | 67 / 265 |  |
| 3,52 | 156,76 | 2018 / 1 |  | 276 | 11 ± 05 | 166 / 412 |  |
| 13,89 | 136,05 | 2018 / 1 |  | 165 | 48 ± 10 | 174 / 303 |  |
| 19,35 | 120,90 | 2018 / 1 |  | 120 | 75 ± 15 | 129 / 216 |  |
| 21,51 | 119,55 | 2018 / 2 | TTOP  6 | 54 | 349 ± 49 | 41 / 92 | West Pacific Ocean |
| 24,84 | 127,89 | 2018 / 2 |  | 95 | 243 ± 32 | 62 / 140 |  |
| 8,13 | 153,42 | 2018 / 2 |  | 139 | 101 ± 16 | 98 / 195 |  |
| 0,45 | 158,31 | 2018 / 2 |  | 106 | 113 ± 19 | 43 / 145 |  |
| -8,82 | 163,09 | 2018 / 2 |  | 208 | 38 ± 08 | 104 / 316 |  |
| -14,49 | 164,67 | 2018 / 2 |  | 49 | 122 ± 31 | 23 / 68 |  |
| -20,02 | 162,63 | 2018 / 2 |  | 137 | 44 ± 11 | 73 / 188 |  |
| -31,52 | 164,44 | 2018 / 2 |  | 145 | 41 ± 10 | 218 / 434 |  |
| -34,24 | 170,86 | 2018 / 2 |  | 37 | 54 ± 31 | 19 / 54 |  |
| -35,07 | 173,95 | 2018 / 2 |  | 50 | 60 ± 25 | 59 / 106 |  |
| -40,06 | 177,94 | 2018 / 3 | TTOP  7 | 89 | 45 ± 15 | 160 / 288 | South Pacific Ocean  Southern Ocean |
| -49,44 | 187,10 | 2018 / 3 |  | 114 | 17 ± 10 | 288 / 355 |  |
| -51,80 | -138,93 | 2018 / 3 |  | 116 | 26 ± 11 | 281 / 457 |  |
| -53,42 | -116,85 | 2018 / 3 |  | 106 | 9 ± 10 | 55 / 155 |  |
| -55,72 | -87,39 | 2018 / 3 |  | 236 | 13 ± 05 | 447 / 688 |  |
| -53,82 | -61,06 | 2018 / 3 |  | 70 | 57 ± 19 | 105 / 212 |  |
| -46,86 | -52,62 | 2018 / 4 |  | 29 | 0 ± 35 | 20 / 48 | Atlantic Ocean |
| -41,05 | -51,85 | 2018 / 4 |  | 108 | 19 ± 11 | 35 / 168 |  |
| -36,20 | -49,27 | 2018 / 4 |  | 86 | 12 ± 13 | 51 / 164 |  |
| -30,83 | -47,75 | 2018 / 4 |  | 105 | 95 ± 18 | 40 / 157 |  |
| -26,32 | -44,39 | 2018 / 4 | TTOP 8 | 64 | 79 ± 23 | 33 / 97 |  |
| -21,66 | -38,47 | 2018 / 4 |  | 143 | 7 ± 08 | 73 / 207 |  |
| -13,78 | -35,76 | 2018 / 4 |  | 83 | 24 ± 14 | 57 / 120 |  |
| -3,10 | -35,67 | 2018 / 4 |  | 118 | 26 ± 11 | 101 / 216 |  |
| 2,17 | -39,07 | 2018 / 4 |  | 70 | 29 ± 17 | 46 / 104 |  |
| 11,63 | -52,57 | 2018 / 5 |  | 174 | 6 ± 06 | 193 / 303 |  |
| 25,43 | -64,81 | 2018 / 5 |  | 74 | 27 ± 16 | 74 / 114 |  |
| 31,00 | -66,51 | 2018 / 5 |  | 45 | 45 ± 26 | 29 / 67 |  |
| 36,82 | -69,08 | 2018 / 5 |  | 66 | 15 ± 17 | 60 / 113 |  |
| 40,38 | -71,15 | 2018 / 5 |  | 14 | 36 ± 74 | 7 / 22 |  |
| 40,16 | -68,31 | 2018 / 5 | TTOP  9 | 27 | 38 ± 41 | 26 / 40 |  |
| 39,73 | -60,88 | 2018 / 5 |  | 81 | 13 ± 13 | 53 / 122 |  |
| 41,15 | -55,67 | 2018 / 5 |  | 28 | 37 ± 39 | 16 / 40 |  |
| 44,10 | -45,91 | 2018 / 5 |  | 52 | 38 ± 22 | 85 / 120 |  |
| 47,68 | -33,53 | 2018 / 5 | TTOP  9 | 33 | 32 ± 34 | 114 / 155 | Atlantic Ocean |
| 51,10 | -22,07 | 2018 / 5 |  | 56 | 18 ± 19 | 39 / 92 |  |
| 51,44 | -16,72 | 2018 / 5 |  | 30 | 33 ± 36 | 22 / 57 |  |
| 50,48 | -8,85 | 2018 / 5 |  | 29 | 35 ± 37 | 14 / 47 |  |
| 51,24 | -4,69 | 2018 / 6 | TTOP  10 | 27 | 38 ± 41 | 12 / 43 |  |
| 52,25 | -11,08 | 2018 / 6 |  | 68 | 0 ± 15 | 9 / 115 |  |
| 55,97 | -10,70 | 2018 / 6 |  | 51 | 20 ± 21 | 68 / 100 |  |
| 58,28 | 3,68 | 2018 / 6 |  | 65 | 31 ± 18 | 58 / 110 | North Sea |
| 58,36 | 10,13 | 2018 / 6 | TTOP  11 | 27 | 112 ± 47 | 15 / 47 | Skagerak  Kattegatt |
| 57,36 | 11,07 | 2018 / 6 |  | 32 | 63 ± 37 | 18 / 53 |  |
| 54,82 | 6,59 | 2018 / 6 |  | 64 | 8 ± 16 | 85 / 201 | North Sea |
| 51,05 | 1,55 | 2018 / 7 | TTOP  12 | 83 | 84 ± 19 | 321 / 929 | Atlantic Ocean |
| 50,07 | -0,75 | 2018 / 7 |  | 248 | 12 ± 05 | 162 / 721 |  |
| 49,27 | -2,57 | 2018 / 7 |  | 157 | 26 ± 09 | 157 / 711 |  |
| 48,14 | -4,69 | 2018 / 7 |  | 139 | 43 ± 11 | 281 / 847 |  |
| 45,99 | -6,71 | 2018 / 7 |  | 299 | 7 ± 04 | 224 / 610 |  |
| 44,45 | -7,96 | 2018 / 7 |  | 357 | 3 ± 03 | 204 / 735 |  |
| 42,85 | -9,09 | 2018 / 7 |  | 314 | 10 ± 04 | 171 / 704 |  |
| 41,10 | -9,14 | 2018 / 7 |  | 395 | 3 ± 03 | 209 / 718 |  |
| 39,30 | -9,40 | 2018 / 7 |  | 376 | 8 ± 03 | 192 / 706 |  |
| -39,63 | 178,64 | 2018 / 3 | AN  7 | 45 | 89 ± 30 | 40 / 83 | South Pacific Ocean  Southern Ocean |
| -53,43 | 194,93 | 2018 / 3 |  | 37 | 107 ± 36 | 44 / 63 |  |
| -52,03 | -116,61 | 2018 / 3 |  | 25 | 41 ± 44 | 32 / 52 |  |
| -55,52 | -83,59 | 2018 / 3 |  | 47 | 0 ± 21 | 76 / 112 |  |
| -45,09 | -52,99 | 2018 / 3 |  | 53 | 19 ± 20 | 45 / 83 |  |
| 37,75 | -61,54 | 2018 / 5 | AN  9 | 73 | 14 ± 15 | 66 / 110 | Atlantic Ocean |
| 37,85 | -51,48 | 2018 / 5 |  | 50 | 0 ± 20 | 43 / 60 |  |
| 52,07 | -17,99 | 2018 / 5 |  | 40 | 0 ± 25 | 33 / 44 |  |
| 50,49 | -6,87 | 2018 / 5 |  | 66 | 15 ± 16 | 31 / 128 |  |
| 53,89 | -8,24 | 2018 / 6 | AN 10 | 73 | 28 ± 16 | 62 / 123 |  |
| 57,34 | 9,59 | 2018 / 6 | AN 11 | 623 | 32 ± 19 | 72 / 88 | North Sea |


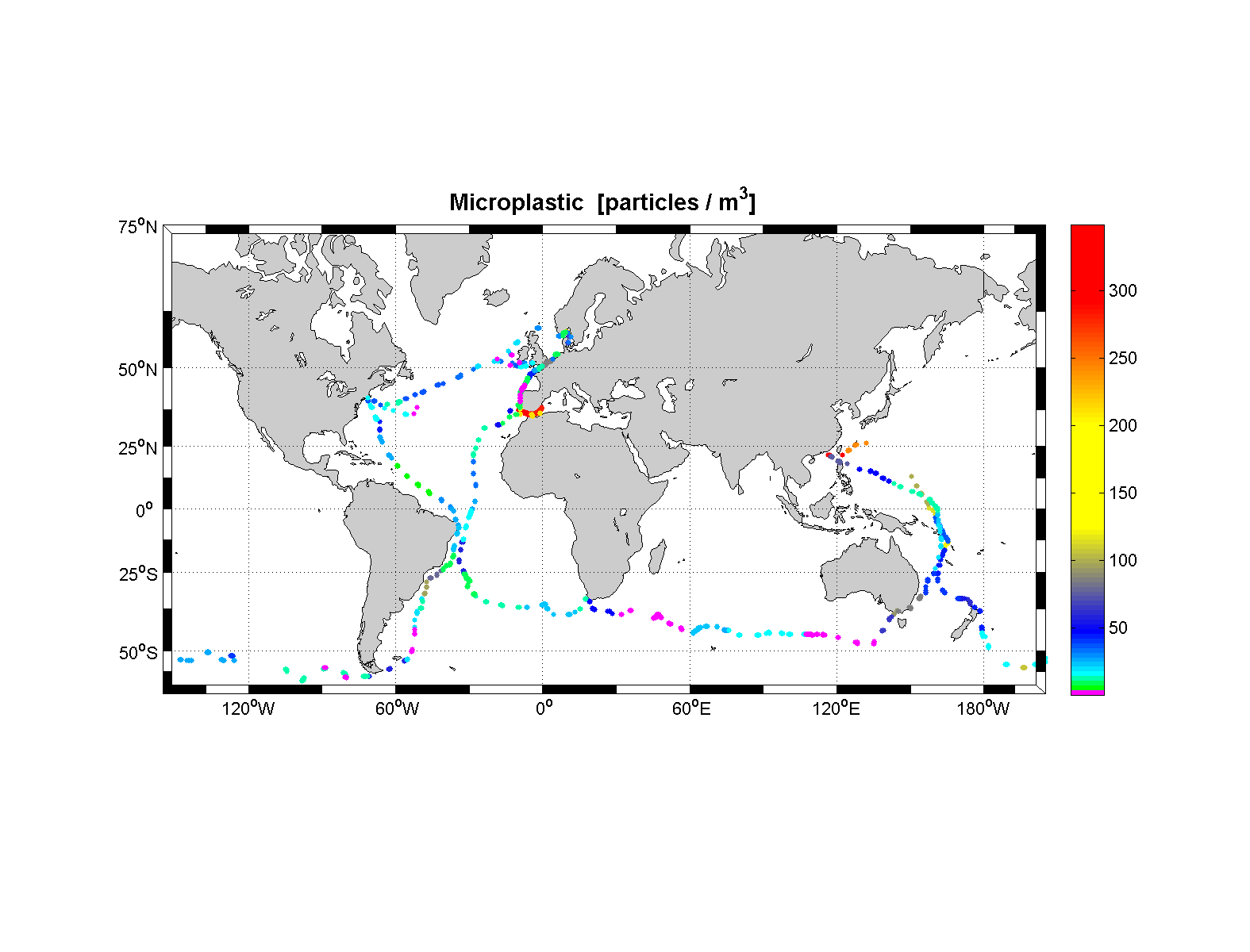


**Figure S2:** This figure represents an alternative way of showing the data in this study. In this map all positions where water was filtered are indicated by equally sized dots, where the color-coding corresponds to the concentration. One can see how many filters were sampled at different locations before being exchanged. The advantage of this map is that all positions are marked, but the same size of the markers makes it more difficult to note the difference in concentration.

**Simulating virtual plastic particles in the Agulhas Current regime around South Africa:**

Using daily average output from the Copernicus Marine Environment Monitoring Service (CMEMS) global ocean 1/12° physics analysis and forecast model (Lellouche et al., 2018), we advected virtual particles representing plastic using the Lagrangian code Parcels (Delandmeter and van Sebille, 2019). We released 1000 particles at 2.6 m depth (3^rd^ model level) randomly distributed in a 1° x 1° box around the two sampling locations at the time of the sampling taken by *Turn the Tide on Plastic* (35.3°-36.3° S, 15.9°-16.9° E for a release at the 25.11.2017 and 36.7°-37.7° S, 1.1°-2.1° E for a release at the 22.11.2017). Using a timestep of 5 minutes, particles were advected backward using the 2-dimensional velocities over a total runtime of 71 days.

Particles released in the influence of Agulhas leakage directly southwest of Cape of Good Hope arrived through the Agulhas Current off South Africa (Figure S2 left). The sampling location thus aggregates particles from a wider region upstream along the African margin. In contrast, the second sampling location (Figure S2 right) is subject to local dynamics only. Particles originate only from nearby.


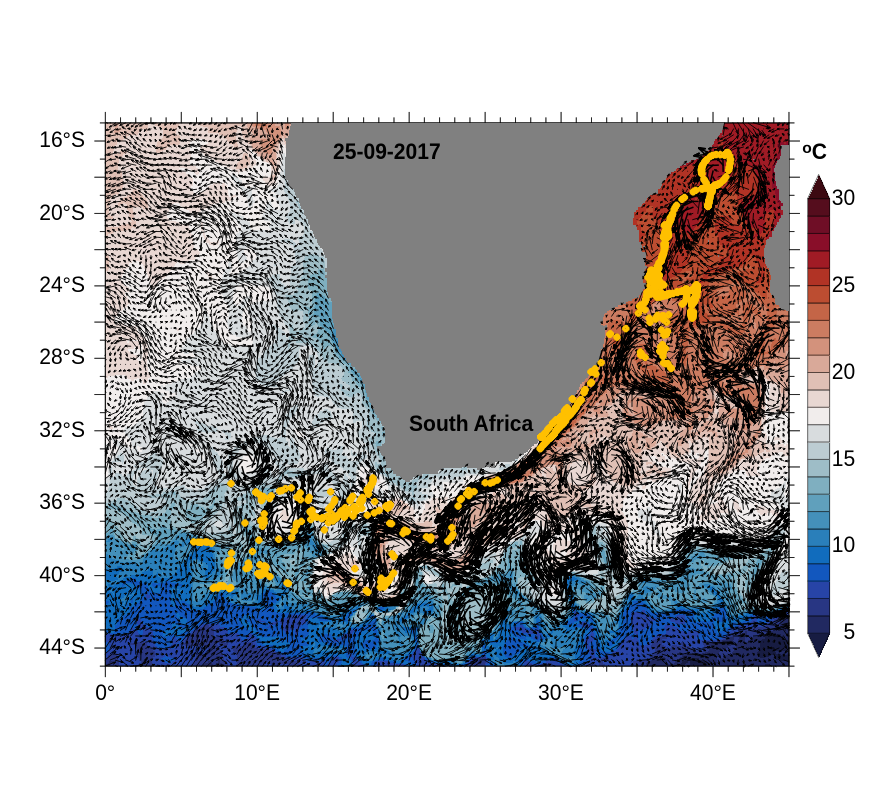

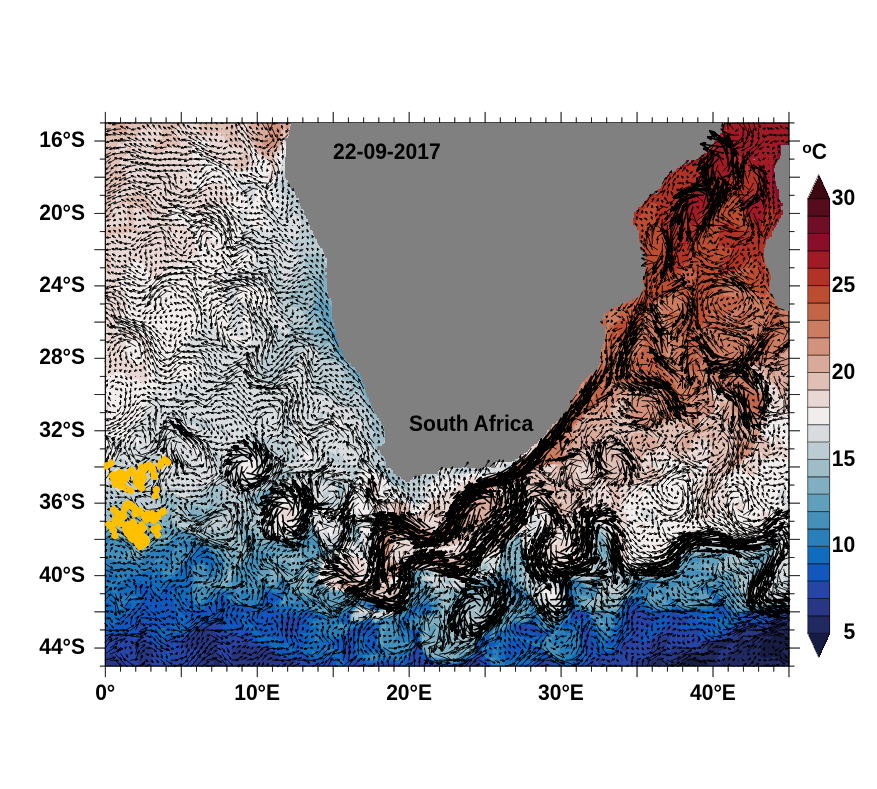


**Figure S3**. Ocean temperatures and currents at 2.6 m depth in the CMEMS global ocean 1/12° physics analysis and forecast model. Shown in yellow are positions of the virtual plastic particles released two months earlier at 35.3°-36.3° S, 15.9°-16.9° E (left) and 36.7°-37.7° S, 1.1°-2.1° E (right). Animations of both dispersal experiments are also provided as supplementary material.

**Animations:**

<https://cloud.geomar.de/s/cKA5s2gKtkZXtMN>

<https://cloud.geomar.de/s/aqEPz7qCfg6sg3i>

**References**

Delandmeter, P., van Sebille, E., 2019. The Parcels v2.0 Lagrangian framework: new field interpolation schemes. Geosci. Model Dev. 12, 3571–3584. doi:10.5194/gmd-12-3571-2019

Lellouche, J.-M., Greiner, E., Le Galloudec, O., Garric, G., Regnier, C., Drevillon, M., Benkiran, M., Testut, C.-E., Bourdalle-Badie, R., Gasparin, F., Hernandez, O., Levier, B., Drillet, Y., Remy, E., Le Traon, P.-Y., 2018. Recent updates to the Copernicus Marine Service global ocean monitoring and forecasting real-time 1∕12° high-resolution system. Ocean Sci. 14, 1093–1126. doi:10.5194/os-14-1093-2018
